# Experiences of community members and researchers on community engagement in an Ecohealth project in South Africa and Zimbabwe

**DOI:** 10.1186/s12910-017-0236-3

**Published:** 2017-12-13

**Authors:** Rosemary Musesengwa, Moses J. Chimbari

**Affiliations:** College of Health Sciences, University of KwaZulu Natal, 1st Floor George Campbell Building, King George V Ave, Durban, 4041 South Africa

**Keywords:** Community engagement, Research ethics, Ecohealth, Community participation, Community involvement

## Abstract

**Background:**

Community engagement (CE) models have provided much needed guidance for researchers to conceptualise and design engagement strategies for research projects. Most of the published strategies, however, still show very limited contribution of the community to the engagement process. One way of achieving this is to document experiences of community members in the CE processes during project implementation. The aim of our study was to explore the experiences of two research naïve communities, regarding a CE strategy collaboratively developed by researchers and study communities in a multicountry study.

**Methods:**

The study was carried out in two research naïve communities; Gwanda, Zimbabwe and uMkhanyakude, South Africa. The multicentre study was a community based participatory ecohealth multicentre study. A qualitative case study approach was used to explore the CE strategy. Data was collected through Focus Group Discussions, Key Informant Interviews and Direct Observations. Data presented in this paper was collected at three stages of the community engagement process; soon after community entry, soon after sensitisation and during study implementation. Data was analysed through thematic analysis.

**Results:**

The communities generally had positive experiences of the CE process. They felt that the continuous solicitation of their advice and preferences enabled them to significantly contribute to shaping the engagement process. Communities also perceived the CE process as having been flexible, and that the researchers had presented an open forum for sharing responsibilities in all decision making processes of the engagement process.

**Conclusions:**

This study has demonstrated that research naïve communities can significantly contribute to research processes if they are adequately engaged. The study also showed that if researchers put in maximum effort to demystify the research process, communities become empowered and participate as partners in research.

## Background

The term community engagement (CE), in health research, describes an array of activities that include dissemination of information, consultation, collaboration in decision-making, empowerment, forming stakeholder partnerships, and seeking guidance from community leaders [1–5]. The working definition used in this paper is that of the Centers for Disease Control and Prevention (CDC) which states that “The process of working collaboratively with and through groups of people affiliated by geographic proximity, special interest, or similar situations to address issues affecting the well-being of those people.” [6]. Community engagement should assure communities and researchers that research conducted is consistent with their sociocultural, political and economic context of where the research is conducted [7].

There are guidance documents [6, 8–11] and literature [12–15] that can assist a researcher in CE but the actual skill and detail of implementation is not prescribed [16]. Researchers have also highlighted the challenges in implementing CE such as power control, conflicting and competing agendas, interfering political/economic interests and considerable time commitment [1–4, 14, 17–19]. They have advocated for capacity building of research teams and communities in order to have meaningful engagement [17, 18, 20, 21]. Capacity building and empowerment of communities and researchers has the potential to assist with understanding research processes. This makes the communities partners in the research process rather than being regarded as just research subjects [17, 18, 21]. This advocacy for capacity building by researchers and sponsors indicates the practical challenges associated with implementing CE in research. One of the areas where researchers have requested for capacity building is development of the skill to extract accurate information from communities with diverse opinions on researched issues [16, 18, 22]. There is some literature [23–25] on how this can be done from the researcher’s perspectives and experiences on CE. However literature on community perspectives on implementation CE is scanty. The studies [18, 20, 22, 26, 27] that described community perspectives generally focused on particular aspects of CE, such as community advisory boards, trust, partnerships, rather than the complete CE strategy. However, the same literature indicates the value of developing culturally appropriate, effective and well-received CE strategies in collaboration with the community [28]. Community members provide a local and unique perspective that ensures that the CE strategy addresses all community concerns before and during a study [14, 21, 29]. However, the perception of what a “community” is complicates data collection on CE. In this study we used the definition of MacQueen [30] that regards community as a group of people with a shared social identity. Thus the term engagement indicates an interactive relationship between a community and a research entity. In this publication stakeholder and community participation is used to mean activities that include physical involvement, generation of ideas, contributions to decision making, and sharing of responsibilities [6]. This is the definition that was adopted for this study.

The purpose of our study was to explore and document the experiences of two research naïve communities and researchers, regarding a CE strategy collaboratively developed and implemented in a multicountry study.

## Methods

A qualitative, case study approach with each study site constituting a case was adopted [31]. This was an appropriate design because both study sites used identical CE strategies thus allowing for direct comparisons between cases.

### The Ecohealth project: MABISA

**Fig. 1:**
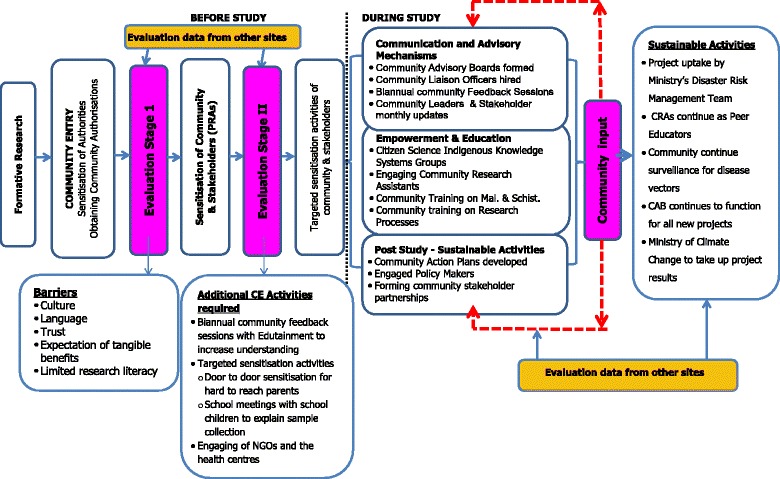
MABISA CE Strategy

This paper describes data collected from an ecohealth study entitled ‘Malaria and Bilharzia in Southern Africa’ (MABISA). The study assessed the impacts of social, environment and climate change on vector-borne diseases in Botswana, South Africa and Zimbabwe, with a view to develop community directed adaptation and mitigation strategies. The study sites were in remote, arid, research naïve and vulnerable environments. The study involved whole communities ie; health workers, schools, policy makers and the ordinary person in the community. The MABISA study used an Ecohealth approach employing various methods including cross-sectional surveys, comparative cross-sectional studies, Participatory Rural appraisal (PRAs) workshops, Geospatial disease and vector Mapping, Focus group discussions and biomedical techniques. During the planning phase of the study, the team systematically collected data on CE strategy (Fig. 1) and implementation. This paper presents data from the Zimbabwe and South African sites only because the Botswana sight had not yet been activated when the documentation of these CE strategies began.

### Study area (case profiles)

The MABISA project was conducted in three wards (Ward 11, 15 and 18) in Gwanda District, Matabeleland South province of Zimbabwe and Ndumo area of uMkhanyakude Health District in KwaZulu-Natal (KZN) province, South Africa. Gwanda district has an estimated 115,778 people of whom 20,225 live in the urban wards. uMkhanyakude is completely rural and has an estimated population of 625,846 people. Both sites are generally arid and characterized by food insecurity and vulnerability to vector-borne diseases (VBD), in particular malaria and Schistosomiasis.They are generally arid and characterized by food insecurity and vulnerability to vector-borne diseases (VBD), in particular malaria and Schistosomiasis [32, 33]. There are many non-governmental organizations (NGOs) and government departments involved in food provision, water and sanitation projects, and distribution of bed nets; and development of irrigation schemes.

Despite these similarities the two communities significantly differ in their social, political and administrative set ups. uMkhanyakude has traditional structures and the delegated “gatekeepers” at the site are the headmen (Nduna) who have jurisdiction over a village. They are accountable to the chiefs, the tribal council and their communities. The MABISA project in South Africa operated in four villages, with one chief and four headmen.

In Gwanda the delegated “gatekeepers” are politically elected councillors. Councillors have jurisdiction over 6-12 villages that constitute a ward. In Zimbabwe, MABISA operated in three wards presided over by three councillors, two chiefs, and 38 village heads. Councillors are accountable to the Rural District Council (RDC), District Administrator and the Member of Parliament in their area. Councillors are, by default, members of every developmental committee in the wards.

### Data collection

Primary data for CE was collected through semi-structured key informant interviews (KII), Focus Group Discussions (FGD), participatory rural appraisal workshops (PRAs), Direct Observation (DO) and Unstructured Interviews (UI). Direct observation and unstructured interviews were used to complement the KII and PRA reports. The MABISA PRAs were intended for community diagnosis and build rapport between the community members and the MABISA study team. The PRA used biannual feedback sessions between the community and the researchers, interviews, focus group discussions and direct observation of community activities using transect walks.

Primary data collection was done by the 1st author (RM) and research assistants. The CE evaluation was led by the two authors on this paper, one of whom (MC) was the PI of the MABISA ecohealth study, and one of whom (RM) was more independent but was the PhD student of the second author (MC). The MABISA principal investigator was the 2nd author of this paper and is included as one of the interviewees.

### Sampling procedure

Key informants were purposefully sampled by selecting several potential key informants (Tables 1 and 2 below) based on their involvement in the CE activities and their knowledge about the MABISA project implementation.

**Table 1:** Distribution of key informants and number of interviews conducted

Key Informants	South Africa #	Number of times Interviewed	Zimbabwe #	Number of times Interviewed
Principal Investigator	1	2	1	2
Researcher Team members	3	2	3	2
Community Research Assistants	5	2	7	2
Councillors	0	0	3	2
Headmen (Nduna)	3	2	0	0
Community Leaders	0	0	1	2
Headmasters	2	1	3	1
Nurses	1	1	1	2
Community Liaison Officer	1	1	1	1
Rural District Council CEO	N/A		1	1
NGO Informant	N/A		1	1
Total number of KII done	28		38

**Table 2:** Distribution of focus group discussion

Focus Group Discussions	South Africa Total # of FGDs	Total # of people in the FGDs	Zimbabwe Total # of FGDs	Total # of people in the FGDs
Community Advisory Board Members	3	25	3	35
Research Team	2	8	2	7
Community members Participants	2	38	3	49
District Health Executive meetings	N/A		1	6
Ministry of Climate Change	N/A		1	5
Total number of FGDs	6		10	

Key Informant Interviews were conducted in three phases; 1) 3 months after the MABISA research team had entered into the study area, 2) after approximately 1 year of study implementation and 3) approximately eighteen months from the time the research team entered into study area. This was done to document how the CE strategy was implemented at various stages of the project. Key Informants included in the analysis provided information at least once.

All respondents in this research provided written consent. A semi-structured interview field guide was developed for in-depth face to face interviews that lasted between 40 min to an hour. The interviews were digitally recorded and/or written down with the participants’ permission.

### Data analysis

The transcriptions of the interviews were thoroughly read by the two authors and a narrative summary was drafted to tease out the data to answer the research questions. The interviews were reread to verify whether the content of the report reflected the most important concepts addressing the research questions. Codes (Table 3) were created from the narrative report and previous literature to identify issues where there was consensus or divergence. Codes were then organised into common themes (shown as first order themes), and the same process was repeated with the first order themes, which were grouped together into second order themes. These were then placed with the matching CE strategy to put them into context. The codes were matched with the quotes associated with each theme and interpretation was done for each CE category.

**Table 3:** Generated Codes

COMMUNITY ENGAGEMENT STRATEGY	2nd ORDER THEMES	1st ORDER THEMES
Formative research, Community sensitisation and approval processes	• Research naivety/inexperience • Flexibility of the CE plan	• Informal and non-specific community entry processes
		• Mistrust of researchers coming into the community
		• Community and stakeholder involvement in study design and planning phase
		• Lack of understanding study by school children and some parents
		• Targeted engagement activities
Communication and Advisory Mechanisms • Community Advisory Boards formed • Community Liaison Officers hired • Biannual community Feedback Sessions • Community Leaders & Stakeholder monthly updates	• Responsibility sharing • Sustained solicitation of opinions of ordinary community • Community input into the CE • Stakeholder involvement	• Community involved in decision making of an appropriate strategy
		• Community selected CAB members
		• CAB requests training
		• CAB took over the communication duties of the CLO
		• CAB & LCL disseminating information to communities
Empowerment & Education • Citizen Science Indigenous Knowledge Systems Groups • Community Research Assistants • Community Training on Malaria & Schistosomiasis • Community training on Research Processes	• Demystification of the research process • Community involvement • Community empowerment • Responsibility sharing	• Community expected direct tangible benefits from MABISA
		• Community requests training in snail and mosquito vector identification
		• Community plans to continue with snail and mosquito vector identification
		• CRAs considered as a “community resource” by the community members
		• Community selected CRAs
Post Study Sustainable Activities • Community Action Plans developed • Engaged Policy Makers • Forming community stakeholder partnerships	• Uptake of MABISA study activities	• Continued surveillance of water points by the CRAs and trained individuals in Gwanda
		• CBMEWS adapted for the Disaster Risk Management Ward Committee
		• uMkhanyakude tribal council requested to use the maps of spatial distribution of Schistosomiasis and infected water bodies
		• NGOs engaged and utilising study data
		• Community Action Plans developed

## Results

The MABISA CE strategy is presented in Fig. 1 for easy reference. In summary the engagement process comprised of 1) community entry activities 2) sensitisation of the community and stakeholders, and 3) implementation and evaluation of the CE strategy (Fig. 1). The study team sought community input soon after sensitisation of the community leaders and stakeholders. This consultation enabled the team to identify barriers and opportunities for CE (Fig. 1). The CE plan allowed the study team to adjust their broad community and stakeholder sensitisation accordingly. For instance, the fact that the community had low research literacy levels meant that during the planed Participatory Rural Appraisals (PRA), a session had to be dedicated to teaching the community on the research process. The CE strategy was also developed during these PRAs. The communities were informed of the broad themes (Fig. 1) of the strategy and the communities chose what they thought would be the appropriate activities for each of the CE categories. In the advisory mechanism category both communities emphasised the need for regular feedback sessions and study activity updates because they felt they did not have enough experience with research and needed to understand what was going on. In the empowerment category they emphasised the need to incorporate more community training on identification of malaria and Schistosomiasis vectors. This was because both sites had access to prevention materials provided by community health workers but they still could not identify the intermediate host snails for Schistosomiasis and malaria vector mosquitos. They also requested training on research processes such as sampling and individual consent process.

One such suggestion was that of relieving the community liaison officers (CLO) of their duties as the community advisory boards (CABs) became more functional. The CLO in uMkhanyakudhe had been referred to the project by the Provincial Health District because he had previously worked as a liaison officer for other NGO projects in the area. The CLO in Gwanda was selected by the study team because he had worked in the district and had extensive experience with both the community members and the clinics. The CLOs initially introduced the study team to the communities and assisted with all initial introductory activities. The communities felt that having the CLOs as well as the CABs was a duplication of activities. The study considered this and the CLOs remained in the study but only in an advisory capacity rather than liaison functions [33]. The communities felt that having the CLOs as well as the CABs was a duplication of activities. The study considered this and the CLOs remained in the study but only in an advisory capacity rather than liaison functions [34].

The perceptions and experiences of the community members and those of the researchers are described below under each category of the CE strategy.

### Experiences and perceptions on the approval processes, community entry, and sensitisation

#### Obtaining approvals and initial introduction to the communities

There was general consensus from both sites that MABISA had surpassed the expectations of the communities in ensuring correct and culturally relevant entry procedures in the absence of any written guidance. The inclusion of the traditional, political and administrative approvals of both sites gave the study authenticity and gained the trust of the authorities. Informants felt that the researchers were sincere as they made great effort to obtain all approvals before commencement.

*“…the study team is very respectful of us and they allow us to instruct them on community protocols and culture and they quickly address issues as needed…I asked them to go back to the RDC for an approval letter and they did so without complaining…now they have their approval and they gave me a copy to see it….most people would just disappear if we ask them to go back to town and seek approval form the RDC…”* Councillor #1 - Gwanda.

The major area where there was divergence of views between community members and the researchers was that for both communities there seemed to be no set standards and/or guidelines where a researcher could refer to. In terms of obtaining the approvals from the authorities the researchers felt that they had paved their own pathway. The community leaders however felt that their informal way of welcoming visitors into their community was easy to follow. The researchers felt that they were unintentionally setting precedence and all other studies were going to be required to follow the MABISA study approval pathway. The community leaders and authorities however did not share the same sentiment as they insisted that even though there is no written guidance on community entry for researchers, there was a societal norm that was informally used for all “visitors” and that was the guideline.

The researchers are quoted here saying:

*“…we went round in circles at first because we had been informed by the CLO that we needed approval from the community first, only to be sent back by the councillor to the Rural District Council before the councillor could give us approval… same with the clinics, we had missed a level of authority (District Health Executive) and had to return only after that committee had approved. For the schools we proactively sought the higher level approvals before we went to the communities…”* Researcher #1 - Gwanda.

*“…we have probably set the standard of how a researcher should conduct community entry as there was no set way and we worked it out as we proceeded with community entry…”* Researcher #2 - uMkhanyakude.

The community leaders however felt that their way of welcoming visitors into their communities was well known and were adequate for a researcher to conduct community entry. They had this to say:

*“…We briefed the community to welcome MABISA ... It is our norm to have an initial meeting to brief the elders so that they give them the go ahead to do their research, for every programme that enters our community...”*CAB Member #2 - Gwanda

*“…it is just not written down in a document but it is known that when a foreign person or company comes into our land, they have to seek permission of the Nduna and the tribal council before they can work in our land…”* Nduna #2 - uMkhanyakude.

Upon further probing why there seemed to be initial hesitation with granting approvals to the study the Ndunas and councillors had this to say ….

“…*When they (researchers) come they are very respectful but after they get the data they just leave unceremoniously…*” Nduna #2 - uMkhanyakude.

*“…I did not quite understand the study when they first came to explain it to me and I felt I needed my community to be part of this so that they benefit as well but I was really scared that if the MABISA people then did something wrong , I was going to be blamed by the community for giving them permission to enter the Ward…I felt better after we discussed it with other councillors at the RDC meeting and I began to see that they were different from the NGO people who come with food or seed maize…”* Councillor #3 Gwanda.

On further discussions of their research experience prior to MABISA,these community leaders gave examples of the population census, demographic health surveys, needs assessments by NGOs and clinic immunisation programme evaluations. In order to allay the fears of the community leaders the researchers took time to explain the differences between communities based participatory research and the other health programmes.

#### Evaluation stage 1

This initial interaction with the communities and the authority figures was analysed resulting in the identification of culture, language, trust and expectation of tangible benefits as the barriers to our CE process. The analysis also identified limited research literacy, lack of community entry protocol/standards, limited community capacity to engage with academics and existence of NGOs as opportunities for community empowerment. These were used to develop a CE strategy that included opportunities for the research team to empower the community (Fig. 1).

During the analysis of the formative evaluation interviews, two major themes, namely research and gatekeeper inexperience became apparent (Table 3). The community leaders were inexperienced with dealing with researchers and involving their communities in research so they could not readily trust. The communities had experience with NGOs and government agencies that basically do evaluations of the food aid programmes. The researchers also had no experience working in these communities and had to learn from the communities before they could implement any research activities. This issue was further explored during the community sensitisation PRA workshops.

#### Community diagnosis and sensitisation (PRA)

All Informants concurred that the MABISA PRA workshops were useful. They all agreed that the workshops helped the community to understand the study and also for the researchers to understand the communities. The Principal Investigator (PI) had this to say after one PRA workshop:

*“…It (PRA) was for us to learn from the community so that we can start working with the community. I think from what I have observe, people have an idea of what our study is about…Most importantly, the success at this workshop has been measured by the study teams’ depth of understanding how this community works…”*PI.

One community leader stated:

*“..The approach was liked because they (MABISA) held community gatherings (PRAS) and introduced themselves to the community …”* Councillor #2 - Gwanda.

Researchers and community members also concurred on the shortcomings of the PRAs. The researchers discovered after the PRAs that some important stakeholders had either not been invited or failed to attend the PRAs.

Some informants had this to say about the PRAs:

“…*our area and some villagers failed to get to the arranged transport sites so early in the morning because the pickup point was still far for me…only one village leader went and he then briefed us on the study after the workshops (PRAs)…”.*

Community member#6 during FGD - uMkhanyakude.

The other important stakeholders who should have been targeted to come to the PRAs were the parents of the primary school children where a parasitology survey was going to be conducted. After the PRAs the researchers stated that they had to carry out targeted sensitisation to ensure that community members understood the study

*“…I only understood MABISA when the research assistants came to my house to explain it. I had gone to the PRA because I thought it was for leaders only…it was a sure sign of respect to actually come to explain it to me before involving my child…”*

Community member#7 during FGD – Gwanda.

The researchers also noted that the PRA method by nature was not going to be enough for complete community diagnosis and sensitisation of large areas and these additional measures of targeted sensitisation in schools and clinics needed to be put in place. [35].

#### Establishing community advisory mechanisms

During the PRAs and sensitisation stages the MABISA research team explained to the community that they wanted to establish a mechanism where they would be continuously appraised on the community’s perception of MABISA in order to remain relevant. Each community was given the option of forming a community advisory board (CAB), having a community liaison officer (CLO) and/or using local community leaders (LCL). The CAB members were elected by the community members at their community meetings, with no interference from the project staff. The CAB members are not paid but given meeting allowances and a small budget to cater for travel expenses and study related activities. The CLO was given a modest allowance. The LCL was only reimbursed transport and food expenses for study related activities.

Communities from both countries chose to form CABs with wide stakeholder representation, to have CLOs and continue having LCL advise the study staff.

At the beginning of the study the PI stated:

*“…At that time (study inception) we had noted the idea of establishing community advisory boards which would be multi-disciplinary in nature encompassing leadership ordinary people and some prominent people in the area and making sure there was a gender dimension infused in it… in that process we only defined what we envisaged the CAB to be like but did not take part in appointing members so we asked the people to organise themselves and tell us who the CAB members will be …”* PI.

One of the Ndunas in uMkhanyakude expressed that:

*“..one of the CAB members is actually the one I call “my eye” because he is also part of the Nduna Security Committee/ Police.. He also gives us MABISA updates at the Tribal council…” *Nduna#2 - uMkhanyakude.

The CAB strategy was also equally supported by the Community Research Assistants (CRAs). They felt that they were “protected” by the CAB:

*“…and when the community has questions about MABISA now the CAB members can answer and are more mature than us with answering questions. The CAB members protect us from the difficult questions…” *CRA #1 - uMkhanyakude.

Community members from both study sites also agreed that the CABs were an appropriate strategy.

During an FGD one community member expressed that:

*“…They (CAB members) are the railway on which MABISA moves in this community…”* Community Member #1.

There was also consensus from all informants on the fact that the CLO position became redundant as the study progressed with the CAB taking over all the duties of that position. The communities felt that the CAB was serving their purpose satisfactorily and having a CLO would be duplication of activities. When one of the CLOs was interviewed, he said:

*“…The CAB members are the eyes of the study in the community…they are like a telecommunications base station taking messages to the community from the study and from the study back to the community…my job here is done and I feel confident that the CAB is carrying out its duties very well…”* CLO#1- uMkhanyakude.

There was also consensus that the study’s continued solicitation for communities’ advice through the CABs, LCL and ordinary community members gave the project legitimacy and showed integrity on the parts of the researchers. Both communities were also satisfied with the fact that their concerns were being taken into consideration and changes implemented as requested or a solution found.

*“…these interviews that you keep holding are producing good results because we see that you are listening to our concerns…we now have an open dialogue with the boss and we even get regular updates from the research team as they come and go every month…” Councillor#3* - Gwanda

#### Empowerment & Education

The study team adopted the following definition of empowerment, *“it is a group-based participatory, developmental process through which marginalized or oppressed individuals and groups gain greater control over their lives and environment, acquire valued resources and basic rights, and achieve important life goals and reduced societal marginalization*” [6].

The PI had this to say about the overall plan on community empowerment.

*“…In order for this (empowerment) to occur we engage the communities fully and we recruit what we call community research assistants who remain with skills (questionnaire data collection, stool & urine collection, sample processing, Geospatial disease & vector mapping) in the society for the future …”* PI.

The PI expressed that the study would involve all age groups within the communities in order to have maximum impact. The employment of the CRAs was meant to empower the young school leavers; the citizen science group was mostly for the much older group of community elders who are the custodians of indigenous knowledge. The PI also expressed that the strategy was not easy to implement as it had to be adjusted as the study progressed. He attributed this to the lack of research experience of both communities.

To achieve this empowerment he expressed that:

*“…we had to strike a balance between training the CRAs to collect credible, usable data and having buy-in for the community training for them (community members) to retain the information imparted and use it for adaptation and mitigation of climate change. If the community does not take up the skills we will impart to them we will have failed to achieve our study goals…the idea is to have the community continuing with these strategies that we will develop with them even in the absence of the CRAs…”*PI.

The PI was specifically referring to the development of community action plans for adaptation to climate change, collection of weather data on indicators of droughts or floods and identification of intermediate host snails for Schistosomiasis and malaria vector mosquitos in their water sources.

#### Engaging community research assistants

The community generally supported the idea of using CRA as a way of achieving community empowerment. However two of the community leaders did not quite understand at the beginning of the study that the MABISA CRA work was not a fulltime paying job and they had this to say during their initial interviews:

*“…I personally do not see any real change in the CRAs life in terms of money, and they spend quite a long time in the month not doing any work. I had thought they would be working everyday even when the study team is not around…”.*

Nduna #3 Interview 1 – uMkhanyakude.

During the second interviews eighteen months after commencement of the MABISA project the same leader had this to say:

*“…I regard them as strictly volunteers because I know the amount of money you give them and it’s not enough for them to consider it a real job…”.*

Nduna #3 Interview 3 – uMkhanyakude.

The other community leader had this to say:

*“…I regard them to be just helping and getting lunch money and I’m not stopping them from getting other opportunities if they are there …”.*

Nduna #1 Interview 2– uMkhanyakude.

The CRAs however were much more appreciative of the exposure they were getting through involvement in actual research and the training they received. They also valued the certificate they received after their training in research methods, fieldwork and research ethics.

This is what they expressed:

*“…My life has changed because people say it when they see me. They see me as an important person now. Some people come to my house to ask about bilharzia and I direct them to the clinic…”* CRA #4 – uMkhanyakude.

*“…working with MABISA has made me believe in myself again and I believe I can still go to university to do a degree in social work…I am now more confident approaching people and talking to them…”* CRA#5 – uMkhanyakude.

*“…I left MABISA recently because I got a job as a security guard at the clinic. It is a permanent government job compared to MABISA. I got the job because they saw me working with the MABISA and they called me for an interview. I answered a lot of health questions correctly because we had been taught a lot by the MABISA team. I however have a family to take care of and cannot continue with the part-time work MABISA gives us...”*

(part-time CRA on his off days at the clinic) CRA#5 – uMkhanyakude.

Similar to the uMkhanyakude site the CRAs were both grateful for the learning experience and certificates but were not so satisfied with the number of days they worked, which translated to reduced allowances.

*“…I did enjoy my work it opened up my eyes to research. I look forward to the certificate to come as it will boost my CV…”* CRA #3 - Gwanda.

*“…People appreciate me more and know that I am a resource person in malaria and bilharzia. I take time to teach my family about identifying mosquito larvae and snail vectors at the rivers. I believe this will help the village in the long run…one person has asked me to diagnose a sick child – they needed to know if they should visit a traditional healer or a go to the hospital and I escorted them to the clinic…”* CRA #4 - Gwanda.

The CRAs were also asked to “teach” other community members about malaria and Schistosomiasis and they demonstrated to their peers that they had knowledge and skills. The CRAs had this to say after the feedback workshops:

*“…They (community) are serious now because before people were not aware on how to prevent these diseases but after the training people have said they will now use mosquito nets and avoid infected water sources...”* CRA#2 -Gwanda.

The community leaders in Gwanda had a different view regarding use of CRAs. They said they viewed the CRAs as a “community resource” and were happy to maintain even after the study ended. They reiterated that the CRAs would continue to collect routine data on malaria vector mosquitos and intermediate host snails for Schistosomiasis and report the information to the local health centres. They also said they would ask the CRAs to go to schools and teach children about malaria and Schistosomiasis transmission so as to prevent infections. This would be like a peer education initiative on malaria and Schistosomiasis. The health workers also mentioned that the CRAs were going to be asked to participate in the malaria indoor spraying programme and the programme on mass drug administration for Schistosomiasis. There were no plans for their remuneration. They said their presence during these activities would help with giving peer education to the school children.

Contrary to uMkhanyakude the Gwanda community leaders said they would not recommend the current CRAs if there were new research studies that came to their area as the current CRAs had already been exposed to research. They felt that the current CRAs had their chance and the next study that came; the community would likely choose a different set of CRAs. This had not been the intention of the project to have the CRAs denied chances to be part of future research studies. They did mention that the MABISA empowerment strategy had set precedence and all studies that will be conducted in their area would be asked to employ at least some local youths for data collection. The community leaders said:

*“…No for now we would not choose them(for any incoming study) since they were in MABISA … The CRAs will be denied entry into other programmes because they already got their chance to shine and learn so any other opportunities for the youth in the area will go to other people...”* Councillor #2 - Gwanda.

*“…I personally favoured the community based research assistants method. MABISA empowered the community by creating part time employment for the youths and it has enabled acceptability of the study as the CRAs are embedded in the community…the next project to come in this area, we will ask them to try and employ locally…”.*

Councillor #1 -Gwanda.

#### Citizen science groups (only done in Gwanda)

One of the MABISA objectives was to develop a community-based malaria early warning system (CBMEWS). The project utilised community elders in each ward to collect data on indicators of weather conditions that may exacerbate malaria. They are able to identify the local plants, animals and astronomical signs to predict rainfall patterns and quantities and relate those indicators to the occurrence of malaria [36]. To motivate participation of these elderly volunteers, the MABISA project will award the participants “citizen science certificates” to recognize their efforts. They too will then become a community resource and will assist the community with weather predictions [37].

The community members who were part of developing the CBMEWS felt honoured that their indigenous knowledge was being put to good use and they learnt the skills of collecting their knowledge in a systematic way. The community members felt they had ownership of the CBMEWS and were happy that they were going to be integrated into the ward level disaster management teams. The community members and the researchers concurred that this strategy was appropriate and would ensure sustainability of the MABISA activities over time.

One of the community members said this during and FGD:

*“…I can finally put my indigenous knowledge to some good use. It’s our first time to have the older generation all involved in one study…everybody is contributing their time and effort to collect data that we will use in the future…”.*

Community member # 5 - Gwanda.

#### Community training & feedback sessions

There were two biannual community feedback sessions at each site in the past 2 years. The feedback sessions had three types of sessions. One session was for the community members to show the study what they understood about MABISA. This was done through drama sessions and poems by the community members, school children and the CRAs on climate change, malaria and Schistosomiasis. The other session was for the research team to provide study updates in the local language through edutainment by poets and actors. The last session was for the study team to address any misconceptions the communities might have and also to update the communities on project activities. [34].

The community felt they had grasped the research information that the study intended to impart within the community.

*“…we have learnt more with MABISA about malaria and bilharzia than what the Ministry of Health has taught us over the years. We now know exactly which snails carry bilharzia and we now know which mosquitos are breeding in any area and which ones cause malaria. We had never known this information before…the health people focus their information on mosquito nets and identifying symptoms but MABISA has taught us everything ….”*

Community Member #2 -Gwanda.

*“…during the feedback session we had the opportunity to make sense of all this research that has been going on….I had no idea that they (researchers) were going to make these maps for us to see where the bilharzia is and which schools are most infected. This information is very useful for us in the villages to know how to protect our children…”.*

Nduna #1 - uMkhanyakude.

*“…the drama was so good…I didn’t understand how the change in weather affects our health like this. I only think of climate change in terms of food production but not in terms of diseases…MABISA has shown me the connection between the two…”.*

Community member #4 -uMkhanyakude.

The CLOs considered the community feedback sessions to have influenced uptake of the MABISA activities to become community activities. CLOs expressed that the community felt respected and honoured to be taught about research process, malaria and Schistosomiasisissues by the research team.

#### Post study sustainable activities

The study team made it clear to the community that they intended to fulfil the ecohealth pillars of knowledge to action and sustainability. The most appropriate way was to facilitate implementation of three main activities, namely; development of community action plans, engagement with policy makers for utilization of the study data for planning interventions and to formation of community stakeholder partnerships that would ensure sustainability beyond the project life span. The community action plans assisted the community in realising their adaptation and mitigation strategies to reduce vulnerability to Schistosomiasis and malaria exacerbated by climate change. The study team has so far presented their work in the departments of health in both countries and have been incorporated in the district health plans [36]. This has shown the value of the study data to the communities. The CBMEWS model developed by MABISA was also presented to the NGOs that work within the same Gwanda community and they have since integrated it with their disaster risk management work [36].

The stakeholders were interviewed about this part of the MABISA CE strategy and there was consensus from all informants that they had not initially understood how MABISA was going to integrate their data with what they had been doing already.

The nurse at one of the clinics had this to say:

*“…honestly I did not expect the project to be so extensive…I have not seen any programme so far that has managed to get my community involved at this level and to maintain the interest of the people this long…I will also adopt their PRA strategy for own clinic programmes before we implement them..”*

School Health Nurse –uMkhanyakude.

*“…we have been trying to find ways in which we can work together with the ministry of health in climate change issues but now MABISA has brought us together because of the CBMEWS which we will use for both malaria and agriculture ….”*

NGO Disaster Risk Management specialist- Gwanda.

The councillor was quoted as saying:

*“…we appreciate the parasitology work being done by the parasitology team. We see the work as simple to implement even after MABISA with the help of the Environmental Health Technicians, so we kindly ask if we can be assisted in coming up with activities that can be used by an ordinary villager…”.*

Councillor # 2- Gwanda.

*“…the CAB will continue to work for the community for any projects that come because they are trained in protecting us from bad researchers now. It is now our advantage that the committee is already well trained…”.*

Nduna #3 - uMkhanyakude.

The communities have appreciated sustainable activities and there has been uptake of MABISA activities by both stakeholders and the community.

## Discussion

The aim of our study was to explore the experiences of two research naïve communities, regarding a CE strategy collaboratively developed by researchers and study communities in a multicountry study. The multicenter study was a community based participatory type of research utilizing an ecohealth approach.

### The context of the study sites

The most significant aspect of the study was the context in which it was being carried out. Both communities admitted that they had limited research experience and that presented an important co-learning and engagement opportunity. Consequently, the researchers had to carry out extensive sensitisation work for both the community and their stakeholders. That motivated the communities to establish a relationship with MABISA through involvement and participation in the project activities. As the relationship between the research team and its communities became more established the contribution of the community to project activities became more apparent. This also shows that research inexperience of communities should not hinder the development of partnerships in research. This also shows that research inexperience should not hinder the development of a fair partnership in research. It simply requires the researcher to proactively, cautiously and conscientiously seek the community’s input in the research process in order to become equal partners. There are other studies in similar African settings [3, 4, 13, 38] that have also demonstrated that it is possible for a research naïve community to effectively engage in research projects. Researchers have however noted that for research naïve communities it is difficult to assess when a community is ready to engage in the research process [38, 39]. Goodman et al. [39] were able to quantitatively measure the changes in the level of knowledge after training community members. They also qualitatively collected information on the perceptions of trainees if the training who reported that they were able to collaborate with researchers after the research training.

### Flexibility of the CE strategy

The fact that the research team was working in sites that were inexperienced in research meant that the research team needed to have a flexible CE strategy that would adapt to both the needs of the community and the needs of the researchers. During the PRAs when it became evident that some key stakeholders had not attended for various reasons the research team and the community had to come up with supplementary activities such as the targeted CE (Fig. 1). This flexibility however meant that the research team had to commit more time, and invested additional financial and human resources. Similar multicentre studies have also indicated the need to adjust a strategy in order to have community members that are fully aware and engaged with the study. Tedrow et al. [38] had to use direct CE consisting of door-to-door canvassing, community meetings, and informal group discussions to ensure that more members of the community were engaged. The flexibility of the CE strategy might also lead to perceived increased scientific rigor in the research plan, and delayed results. [26, 40].

### Communication and advisory mechanisms

There was consensus amongst all the informants that the communication and advisory mechanisms were appropriate for the communities because the community felt respected to be involved in decision making. This perception of sharing responsibility was a direct result of the researchers deciding that major aspects like selection of CRAs, Citizen Science IKS groups and CAB members were done by the community without interference of the project staff. The community was also asked to disseminate information at local community meetings outside the MABISA scheduled meetings so as to keep the community members informed of the study activities. All the informants indicated that this was an appropriate strategy for these communities. The fact that the study team did not involve themselves in the selection of community members for these activities ensured that they did not get entangled in any historical power dynamics. This gave the community leaders a sense of responsibility for their contribution to the CE strategy.

Dickert and Sugarman (2005) [29] stress that sharing responsibility with community members confers a degree of moral responsibility on communities for the research and they too became an accountable partner in the process. Similar studies [3, 13, 23, 41–43] have reported that sharing of responsibilities can build trust and legitimacy in a research partnership. Furthermore it has been shown that community engaged research that emphasizes responsibility sharing and collaboration, leads to sustained engagement with the communities [26, 44]. The same authors also caution that even if the research requires CE, sharing responsibilities and decision making, this does not absolve the researchers of their responsibility to carry out rigorous research.

The communities also acknowledged the researchers’ continuous solicitation of opinions of ordinary community members as genuine engagement that empowered them. The research team reiterated that the study needed to be consistently appraised on how the community wanted to be engaged. The researchers envisaged that this would ensure that the community would assume ownership of the CE strategy. In studies where, communities are mostly informed of what is going on and much less asked to make any decisions or to participate, the engagement is much less effective [3, 45, 46]. Kolopack et al. [45] explains that continued solicitation for advice ensures that the community influences the engagement process. This opportunity to express themselves and choose which strategy they want would take care of the limitations of the community advisory mechanisms that MABISA utilised. Literature points out the need to strike a balance on the dual functions of CABs in research of advancing the research agenda and also protecting the community [47]. This was noted by the informant who likened the work of the CAB to a *“telecommunications base station”* taking messages to the community from the researchers and vice versa. This shows that even though the MABISA communities perceived CABs as appropriate, researchers in other contexts still have to carefully consider community input on the advisory mechanism that best suits them [47]. The major challenge to the CAB model for other studies [29, 47] has been identifying community members with legitimate interests in protecting communities.

### Empowerment & Education of the communities

The MABISA study demonstrated that where there is authentic community engagement in a research naïve site there is demystification of the research process leading to increased participation. Informants concurred that they initially had no idea what the study was about but the level of engagement by MABISA and efforts to educate the community it was highly informative and empowering. The community demanded more training, an indication that they perceived the study as beneficial to them. They concurred that the MABISA education plan was empowering them at all levels in the community. The research team had recognised that the low research literacy in the communities represented the opportunity for engagement (Fig. 1).

The findings from the MABISA study are significant as they suggest that even though low research literacy may present unexpected challenges, it should not be viewed as a barrier but an opportunity for engaging through educational activities. The study trained a diverse group of community members on data collection, specimen collection and processing. The study team invested time and resources to training ordinary members to collect credible research data. This was high level engagement for the MABISA project where the researchers made their work and their processes comprehensible to laypersons. The Gwanda community has committed to utilise the citizen scientists even when the MABISA study has ended showing the appropriateness of this CE strategy to the community. Similar work of increasing research literacy and competency through citizen science, training in research methods and participatory activities has been done [25, 39, 48–50] and reported to have empowered communities. Training citizen scientists is a transformative that enhances formation of equitable community-academic partnerships [39]. However from the MABISA study experience researchers should engage the community first and find out how much scientific work they can participate in and they should be given a chance to choose the people they feel are most capable to commit to being trained. Dickinson et al. [50] also points out that citizen science, though good presents analysis-related challenges such as sampling bias, observer variability, and detection probability. Citizen science also requires substantial investment in financial resources and time to train citizens to the point where they can collect credible research data.

### Post study sustainable activities

The MABISA study made deliberate efforts to ensure that the skills and knowledge imparted into the communities would remain relevant to the communities even after the study had ended. The Gwanda community appreciated the effort of ensuring that the contributions of the IKS citizen science groups in developing a CBMEWS would be integrated into the district level disaster risk plans [36]. This created a platform for further collaboration between the departments of agriculture, health, NGOs and the community at decision making level. The community noted that MABISA had made it possible for them to form mutually beneficial stakeholder partnerships.

In uMkhanyakude, the community was particularly interested in the maps showing spatial distribution of Schistosomiasis and malaria as they noted that they would ordinarily not have access to such maps. They felt that they now had their own local maps which they could use for planning local activities [51]. This suggests that the community had had limited skills for negotiating for locally relevant health information from the government or the NGOs that came to their area. The MABISA team trained them how to collect simple GPS data using their phones and plot a simple map of where intermediate host snails for Schistosomiasis and malaria vector mosquitos had been spotted. The ability of a community to identify exactly what interventions they want to sustain is a skill in itself. Development of new skills of data collection and interpretation could possibly be used as measures of success of a CE strategy [14, 26].

### Divergent views between the two studies communities

Contrary to the uMkhanyakude community who did not seem to value much their CRAs, the Gwanda community viewed its CRAs as a “community resource”. This is probably because Gwanda viewed the training that the CRAs received as superior and they saw it as a change in social status and they did not want to lose the knowledge they had gained through the CRAs. The other factor could be that the community wanted the CRAs to continue with surveillance activities for malaria and Schistosomiasis. This would relieve the environment health worker who had to cover three wards and would not be able to continue surveillance work at the same frequency as done by the MABISA team. They also had asked them to continue to become peer educators for the school children and teach them about the dangers of swimming in contaminated water bodies. This was not the case with uMkhanyakude that had a health system that was more resourced and accessible than the Gwanda community and hence might not easily appreciate the services of the CRAs.

The other difference was that the uMkhanyakude community also did not view the MABISA work as a significant opportunity for the CRAs not to be involved in other opportunities that may have arisen within the community. On the contrary the Gwanda community considered the MABISA CRA work as very significant. However, the leaders communicated that they would not be chosen for further opportunities as they were many young people in need of similar research exposure as well. The explanation could possibly be that Zimbabwe as a country does not have a social grant programme like South Africa such that any form of allowance is considered significant to an unemployed youth. In South Africa one of the Ndunas actually said they considered the field allowances MABISA gave them as “lunch money” because they did not work fulltime and the amount would be dependent on the number of field days worked per month. He did not see MABISA as a financial opportunity but only as an educational experience.

### Limitations

Whilst the experiences and CE strategies were found to be appropriate for this 3 year study, they may not be directly relevant to all community based participatory studies, or to other study designs. The multicentre CE strategy described in our paper will likely be most appropriate for long-term studies lasting more than 1 year. In shorter term studies both the CE and the evaluations of CE activities will also need to be carefully timed to allow them to feed into the overall CE strategy. In study areas where the communities are familiar with research activities and concepts, the capacity building components might not need such emphasis, and studies with limited funding might not be able to carry out such elaborate CE and evaluations.

The Ecohealth approach to research is very participatory in nature and this might have had an influence on the outcomes of the CE described in this paper. Ecohealth is a methodology that intrinsically has community and stakeholder participation as one of its pillar principles. In studies where community and stakeholder participation are not one of the core methodologies or requirements perhaps a very elaborate CE plan might not be as necessary. Researchers might need to streamline activities to suit their core methodologies.

Whilst we remain confident of the findings, having the PI (MC) as a co and senior author, and involved in the research and analysis might have influenced our interpretation of the process and it’s outcomes. However, we believe the findings have relevance to the literature on CE in multicentre studies.

## Conclusion

This study has demonstrated that a community’s views and perceptions play an important role in shaping the CE process. The study also demonstrated that when a study continuously evaluates its CE there will be less divergence of views between researchers and communities; and the CE process becomes mutually beneficial. The views of the community allowed the researchers to have insights into what the community expected and how they wanted the engagement process to proceed. This led to greater comprehension of the study, increased participation, ensured sustained interest, empowered community members to interact with its stakeholders as partners and ensured the knowledge and skills learnt were adopted by the community and its stakeholders.

## Abbreviations

CAB: Community Advisory Board
CBMEWS: Community-based malaria early warning system
CE: Community Engagement
CHW: Community Health Workers
CLO: Community Liaison Officer
CRA: Community Research Assistant
DA: District Administrators
LCL: Local Community Leaders
MABISA: Malaria and Bilharzia in Southern Africa
NGOs: Non-governmental organizations
PI: Principal Investigator
RDC: Rural District Council
VBD: Vector-borne Diseases
